# Unexplained recurrent arthritis as presenting sign of hereditary autoinflammatory syndromes

**DOI:** 10.1186/1546-0096-9-S1-P25

**Published:** 2011-09-14

**Authors:** F Zulian, G Vigo, F Vittadello, I Ceccherini, L Obici, G Martini

**Affiliations:** 1 Rheumatology Unit, Dept. of Pediatrics, University of Padua, Italy; 2 Molecular Genetics Laboratory, G. Gaslini Institute, Genova, Italy; 3 Genetics Laboratory, IRCCS San Matteo, Pavia, Italy

## Background

Hereditary autoinflammatory syndromes (HAS) are a group of rare monogenic inherited conditions characterized by recurrence of symptoms of whom fever is the most frequent.

## Aim

To analyze the prevalence of recurrent arthritis in a cohort of patients with HAS.

## Methods

We conduced a retrospective study on 300 patients with periodic and recurrent fevers referred to a single tertiary center in 17 yrs of activity. Patients with recurrent arthritis were identified and fully investigated. On the basis of the clinical features and genetic tests, patients were divided into two groups: 275 PFAPA syndrome and 25 HAS.

## Results

Patients with monogenic HAS have a later disease onset (63.4 vs 27.9) and a higher frequency of abdominal pain (60% vs 16.4%), headache (32% vs 7.35%) than PFAPA. Recurrent arthritis was found in 9 HAS (36%), none in PFAPA. Interestingly, four of these 9 patients had unexplained recurrent arthritis as unique manifestation of the disease for a long time. Arthritis consisted in episodes of joint swelling, pain and limitation of movement involving, asymmetrically, large joints. In three patients most episodes occurred after infections. Attacks resolved spontaneously in few days but during disease-free intervals acute-phase reactants (CRP and SAA) were still abnormal. Genetic analysis showed heterozygous missense changes of the MEFV gene(A289V, P369S) in two patients and of MVK (V250I and G336S, I268T) in the other two.

## Conclusions

Unexplained recurrent arthritis may be the unique manifestation of a monogenic HAS and should be added to the list of possible differential diagnosis of the so called “palindromic arthritis”.

